# Mechano-induced homotypic patterned domain formation by monocytes

**DOI:** 10.21203/rs.3.rs-3372987/v1

**Published:** 2023-09-22

**Authors:** Denis Wirtz, Wenxuan Du, Jingyi Zhu, Yufei Wu, Ashley Kiemen, Zeqi Wan, Eban Hanna, Sean Sun

**Affiliations:** Johns Hopkins University; Johns Hopkins University; Johns Hopkins University; Johns Hopkins University; Johns Hopkins School of Medicine; Johns Hopkins University; Johns Hopkins University; Johns Hopkins University

## Abstract

Matrix stiffness and corresponding mechano-signaling play indispensable roles in cellular phenotypes and functions. How tissue stiffness influences the behavior of monocytes, a major circulating leukocyte of the innate system, and how it may promote the emergence of collective cell behavior is less understood. Here, using tunable collagen-coated hydrogels of physiological stiffness, we show that human primary monocytes undergo a dynamic local phase separation to form highly regular, reversible, multicellular, multi-layered domains on soft matrix. Local activation of the β2 integrin initiates inter-cellular adhesion, while global soluble inhibitory factors maintain the steady state domain pattern over days. Patterned domain formation generated by monocytes is unique among other key immune cells, including macrophages, B cells, T cells, and NK cells. While inhibiting their phagocytic capability, domain formation promotes monocytes’ survival. We develop a computational model based on the Cahn-Hilliard equation of phase separation, combined with a Turing mechanism of local activation and global inhibition suggested by our experiments, and provides experimentally validated predictions of the role of seeding density and both chemotactic and random cell migration on domain pattern formation. This work reveals that, unlike active matters, cells can generate complex cell phases by exploiting their mechanosensing abilities and combined short-range interactions and long-range signals to enhance their survival.

## Introduction

The human body is composed of tissues that span a wide range of stiffnesses^1^, ranging from 11 Pa (intestinal mucus) to 20 GPa (cortical bone)^2^. Tissue stiffness is also altered during aging^3–5^ and under various disease conditions, such as cancer^6–9^ and inflammation^10^. Mesenchymal and epithelial cells have evolved complex molecular mechanisms to sense and respond to these different environmental mechanical cues to differentiate, signal, and migrate. How immune cells respond to mechanical cues has received significantly less attention. In particular whether and how monocytes, which are pivotal components in the innate immune response, respond to microenvironments of different stiffness is not well understood^11^. Microenvironmental molecular signals can differentiate monocytes into monocyte-derived macrophages and dendritic cells, which enables them to orchestrate innate and adaptative immune responses^12,13^. Upon infection, inflammation or tumorigenesis, classical (CD14^+^CD16^−^) monocytes that mature in the bone marrow and emigrate to the peripheral blood constantly traffic between blood vessels and soft tissues^14^, making them more likely to encounter complex micro-environments of different stiffness.

Imaging of pre-cancer and tumor tissues has revealed spatially heterogeneous immune cell hot spots – dense aggregates of immune cells - in the stomal region of precursor lesions, such as PanIN in the pancreas, as a reservoir for future infiltration^15,16^. Accumulating studies also suggest that homotypic immune cell aggregation or domain formation is a physiologically relevant process that plays a vital role in pathogen clearance and cancer metastasis for B cells^17,18^, neutrophils^19,20^, dendritic cells^21^ and monocytes/macrophages^22^. Cells connect with neighboring cells and their milieu by forming molecular links driven by adhesion molecules, including cadherins, selectins, integrins, Ig-like adhesion molecules, and mucins^23^. Integrins are highly expressed on the surface of monocytes, which have been found to be essential for the tethering and rolling process of monocyte extravasation and as one of the major mediators for cell-matrix adhesion via focal adhesion complex^24–26^. Whether and how matrix mechanical properties promote or modulate immune cell aggregation and domain formation is unclear.

The observed immune cell aggregates are reminiscent of dynamic phases observed in active matter systems^27^. In particular, flocking transitions seen during collective movement of fish and birds also produce high density aggregates^28,29^. More recent theoretical work has revealed rich phase behavior of self-propelled particles and their underlying microscopic interactions^30–33^. Different from flocking, which are governed by local short-range interactions between self-driven particles, cells feature both short-range interactions and long-range signals, and therefore potentially can generate complex behavior. Unlike active matter systems, cells can also respond to differences in matrix stiffness (mechanosensing), secrete factors, thrive to survive and proliferate, and move along local chemotactic gradients. Moreover, cell movement is distinct from propelled particles, and are instead described by persistent random walks (PRW)^34,35^. These distinct aspects led us to hypothesize that immune cells would collectively reveal new dynamic phases and behaviors.

In this study, we find that physiological and pathological matrix stiffness can spontaneously trigger monocyte homotypic patterned domain formation via β2 integrin over-expression to promote long term cell viability. We propose a phenomenological model based on the Turing mechanism and Cahn-Hilliard equation for phase transitions ^36,37^ which incorporate activation of β2 integrins as a local activator and self-inhibitory soluble factors secreted by aggregated monocytes as global inhibitors, which simulate the patterned domain formation observed on collagen-coated hydrogels. Effects of cell seeding density and cell chemotaxis *vs*. random migration on monocyte homotypic patterns are predicted by computational simulations and validated in corresponding experiments.

## Results

### Soft matrix induces multicellular domain formation of monocytes

**Figure 1:**
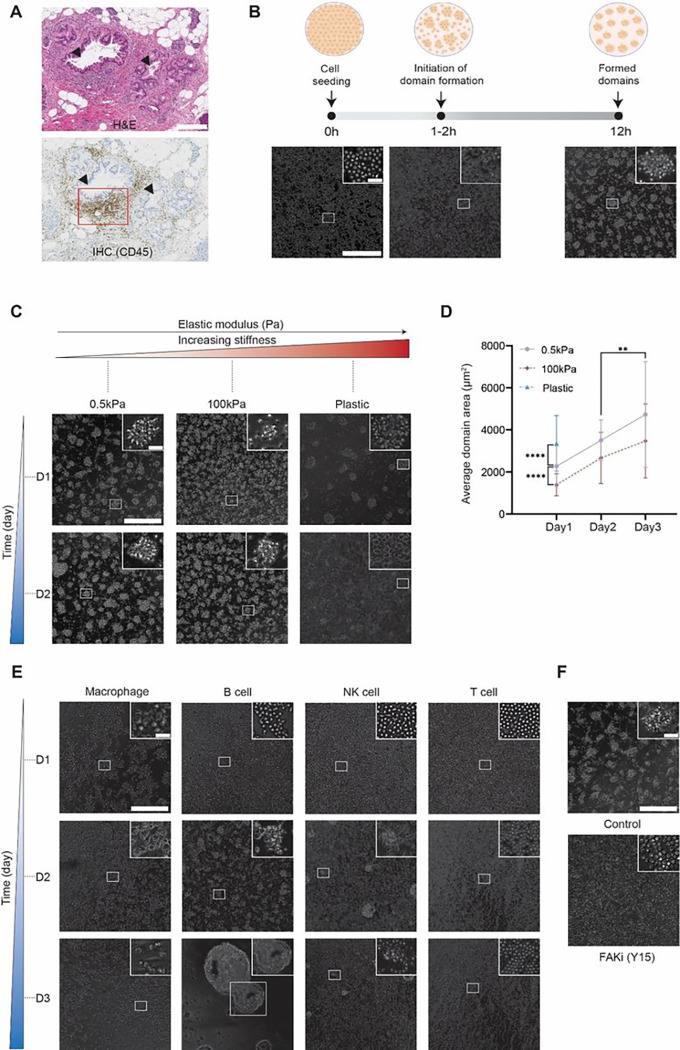
Mechano-induced homotypic domain formation of human primary monocytes on substrates of different stiffness. (**A**) Representative sections stained with hematoxylin and eosin (top, H&E) and CD45 (bottom, leukocyte common antigen) showing an immune hot spot in a human pancreatic tissue containing a precursor lesion (PanIN). The large gland on the left is ‘hot’ with aggressive accumulation of leukocytes (highlighted in red box) while the small gland on the right is relatively ‘cold’ with minimal immune cell infiltration. Scale bar, 300 μm. (**B**) Freshly isolated human primary classical (CD14^+^CD16^−^) monocytes formed compact multicellular domains when placed on a 0.5 kPa collagen I-coated substrate. Representative phase-contrast images were obtained with a 20X objective at 0h, 2h, and 12h. Monocytes spontaneously form pre-domain spots around 1–2h after initial seeding. After 12h incubation, these spots progress to highly patterned domains (see Supplementary Video 1 for domain formation process). (**C**) Representative phase-contrast images of monocytic domain formation on (soft) 0.5 kPa, (stiff) 100 kPa and (hard) plastic collagen I-coated substrates obtained via a 20X objective on day 1 and day 2. (**D**) Average areas of domains formed by monocytes on substrates of different stiffness. Monocytic domains did not form on collagen-coated plastic substrates and were therefore not characterized on day 2 and day 3. Enlarged domain areas were observed on 0.5kPa and 100kPa from day 1 to day 3, which were potentially caused by partial coalescence of single monocytes into established domains or “flattening” of multi-layered domain structure. (**E**) Representative phase-contrast images of macrophages / B cell / NK cell / T cell domain formations on 0.5 kPa soft substrate obtained on day 1, day 2 and day 3. None of the lymphocytes initiated homotypic aggregation for the first 24h like monocytes’ quick response to mechanical cues of the substrate. Macrophages, however, displayed homotypic aggregation on day 1 but failed to maintain. Representative day 1 images were taken with 10x images while day 2/3 images were taken with 20x objective. (**F**) Inhibition of focal adhesion kinase using 20 μM Y15 abrogated domain formation. Representative 20x images were taken on day 1. Scale bars, 300 μm (insets, scale bar, 30 μm). Unpaired student’s t-test was used for statistical analysis. Results are presented as mean ± standard deviation.

The focus of the paper is to explore the emergence of collective physical phenomena resulting from monocytes exposed to substrates of different stiffnesses. But we first place our work on immune cell aggregates in a larger biological context. Figure 1A shows a localized high density of leukocytes in human pancreas tissue. Two consecutive 5-μm thick tissue sections were respectively stained with hematoxylin and eosin (H&E) to enable visualization of the pancreatic microanatomy (top section) and immuno-cytochemically stained for the cell-surface antigen CD45 to label all leukocytes (bottom section). The function and mechanism of such leukocyte-rich domains remain unclear. To investigate the minimum necessary conditions for leukocytes to form such aggregates *in vitro* and assess the potential role of matrix stiffness, we placed freshly isolated human classical (CD14^+^CD16^−^) monocytes on collagen-I coated polyacrylamide gel substrates of stiffness 0.5 and 100 kPa, as well as standard collagen-I coated cell culture plastic substrates (~ 5 GPa). These substrates were chosen to mimic the range of tissue stiffness encountered by monocytes during disease progression of soft tissue such as the transition from the relatively soft microenvironment of the normal human breast to the comparatively stiff microenvironment of breast cancer^38^. All substrates in this work were coated with a saturating amount of collagen I – the main constituent of the extracellular matrix in stromal spaces – to keep biochemical ligand presentation to the cells constant.

Following seeding as a monolayer on 0.5 kPa collagen-I coated substrates, we observed that monocytes remained mostly featureless for an “incubation” time of 1–2 h. This incubation time for domain formation, during which monocytes remained a monolayer, was remarkably consistent across donors and across different wells for a given donor (Supplementary Fig. 1). Then a rapid phase separation occurred, and multicellular aggregates formed (Supplementary Video 1). In the first few hours post-transition, the borders of these aggregates were relatively diffuse. After ~ 12h, these aggregates annealed and formed well-defined spatially patterned muti-layered aggregates, referred below as “domains” (Fig. 1B). In contrast, domain formation of monocytes placed on 100 kPa (stiff) substrates experienced a significant delay and resulted in the development of irregular domains on day 1 (Fig. 1C). On collagen-I coated plastic culture dishes (~ 5GPa), monocytes did not show long-lasting domains. Monocytes placed on this stiff substrate formed highly transient domains, whose average size was significantly larger compared to those on the 0.5 kPa and 100 kPa substrate on day 1 (Fig. 1D). These domains collapsed after day 2 (Fig. 1C).

We asked if stiffness-mediated patterned domain formation was common among immune cells. We placed either primary human monocyte-derived (M0 naïve) macrophages, B cells, NK cells or T cells on 0.5 kPa substrates and assessed potential phase separation. When freshly isolated from human peripheral blood, none of these lymphocytes could initiate ordered domain formation when exposed to mechanical cues within the first 24h, suggesting the unique ability of monocytes to form patterned aggregates via mechano-sensing. Macrophages aggregated instantly after seeding, but formed continuous cell clusters instead of spatially distinct patterned domains. Importantly, the majority of these macrophage aggregates collapsed and disappeared on day 2 and individual cells with mesenchymal morphology were observed strongly attached to the substrate on day 3 (Fig. 1E). Over a longer timescale, T cells remained as a monolayer for three consecutive days, while NK cells only formed small, sparsely distributed, diffuse clusters. For B cells, which are known to aggregate following stimulation^17^, we observed patterned domain formation after 48h, but these domains subsequently merged, prompting a macroscopic phase separation. These large aggregates even partially detached from their collagen-I coated substrates (Fig. 1E). Finally, after long-term activation with IL2 cytokine before seeding on 0.5kPa substrates, both B and NK cells aggregated instantly to subsequently undergo macroscopic phase separation (Supplementary Fig. 2). T cells activated with IL2 and CD3/CD28 Dynabeads mostly remained as a monolayer in co-existence with a few small clusters (Supplementary Fig. 2). In sum, only freshly isolated monocytes formed long-lived, highly patterned homotypic aggregates following exposure to a soft substrate.

To further support the hypothesis that monocyte domain formation was induced via cell mechano-sensing, we treated the monocytes with an inhibitor of the major mechano-sensing protein focal adhesion kinase (FAK)^39–41^. Monocytes did not exhibit any evidence of domain formation (Fig. 1F, Supplementary Video 2). This further supports that mechano-sensing plays an indispensable role in the process of domain formation by monocytes. Together these results suggest that monocytes can spontaneously form regular multicellular domains, a process prompted by the stiffness of the underlying matrix.

### Phase separation promotes cell survival and inhibits phagocytosis

**Figure 2:**
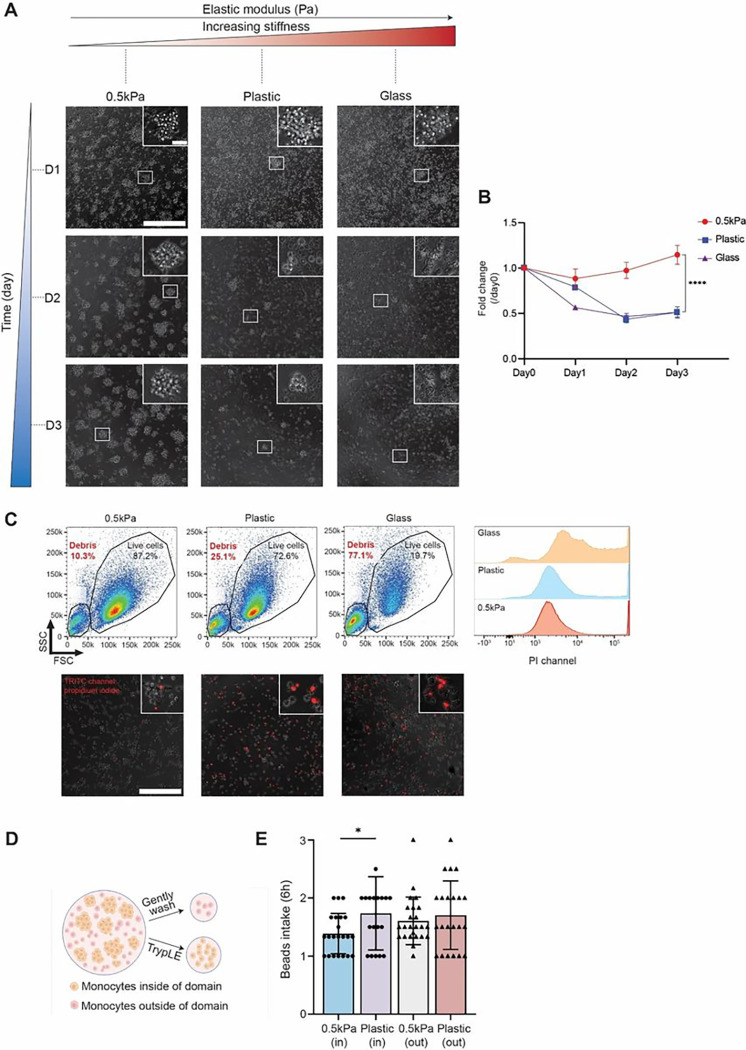
Effects of homotypic domain formation on monocyte viability and phagocytosis. (**A**) Domain formation and long-term maintenance on collagen I-coated 0.5 kPa, plastic and glass substrates. Representative phase-contrast images were taken with a 20X objective on days 1, 2 and 3. (**B**) Fold change in PrestoBlue signal normalized to day 0 when seeded. Significant cell death was observed for monocytes seeded on collagen I-coated plastic and glass substrate with dramatically decrease of PretoBlue signals compared to day 0. In the meantime, monocytes seeded on collagen I-coated 0.5 kPa substrate maintained a similar level of metabolic activity during the 3-day monitoring window, suggesting long-term maintenance with negligible effects from proliferation. (**C**) Flow cytometry results of monocytes harvested from collagen I-coated 0.5kPa, plastic and glass substrates on day 3. respectively. More cell debris and propidium iodide positive cells (died cells) were observed when monocytes were seeded on plastic and glass substrate. Representative Immunofluorescence images taken with 20x objective on different substrates were shown via merging phase contrast channel and PI channel. (**D**) Illustration cartoon that distinguishes monocytes inside and outside domains. (**E**) Characterization of phagocytosis capabilities of monocytes inside and outside of domains, measured via quantification of GFP-positive Carboxylate-modified polystyrene beads (1 μm) intake after 6 hours. Scale bars = 300 μm (insets, scale bar = 30 μm). Unpaired student’s t-test was used for statistical analysis. Results are presented as mean ± standard deviation.

Next, we investigated possible functional outcomes of monocyte domain formation. Monocytes seeded on 0.5 kPa collagen-coated substrate showed significantly higher viability than monocytes seeded on collagen-coated plastic and glass substrates within a time frame of 72h, for which collapsed domains and cellular apoptosis were clearly observed at days 2 and 3 (Fig. 2A). Quantitative live cell viability assay via PrestoBlue showed a dramatic decrease in signal from monocytes on collagen-I coated plastic and glass substrates in contrast to monocytes on collagen-I coated 0.5 kPa substrate. This result indicates an underlying functional connection between the ability of monocytes to form and maintain domains and their viability (Fig. 2B). Monocytes were also harvested after 72h from different substrates and analyzed using flow cytometry. While debris remained at a relatively low value of 10.7% for monocytes harvested from 0.5 kPa substrate, elevated proportions of debris were observed on stiffer substrates, 25.1% for plastic and 77.1% for glass (Fig. 2C). Finally, more monocytes positive for propidium iodide were observed through immunofluorescence imaging and flow cytometry, which worked as extra validations of reduced cell viability on non-physiological substrates (Fig. 2C). In contrast to freshly isolated monocytes, the majority of activated immune cells died after three days in culture (Supplemental Fig. 2).

To study whether substrate stiffness and domain formation had any impact on the phagocytic ability of monocytes, we evaluated the number of internalized latex beads. We distinguished monocytes located within the domains as “insiders” and those outside the domains as “outsiders” (as illustrated in Fig. 2D). On the 0.5 kPa substrate, insiders and outsiders exhibited similar levels of bead phagocytosis, with insiders averaging 1.6 ± 0.4 particles per cell and outsiders averaging 1.4 ± 0.3 particles per cell. Likewise, on the plastic substrate, there was no significant difference in phagocytosis ability between insiders and outsiders (Fig. 2D). However, “insiders” on 0.5 kPa substrate displayed a small but statistically significant lower phagocytic activity compared to those on plastic substrate, which suggests a negative correlation between monocyte viability and phagocytosis.

### Matrix stiffness modulates β2 integrin expression, which mediates homotypic aggregation

**Figure 3:**
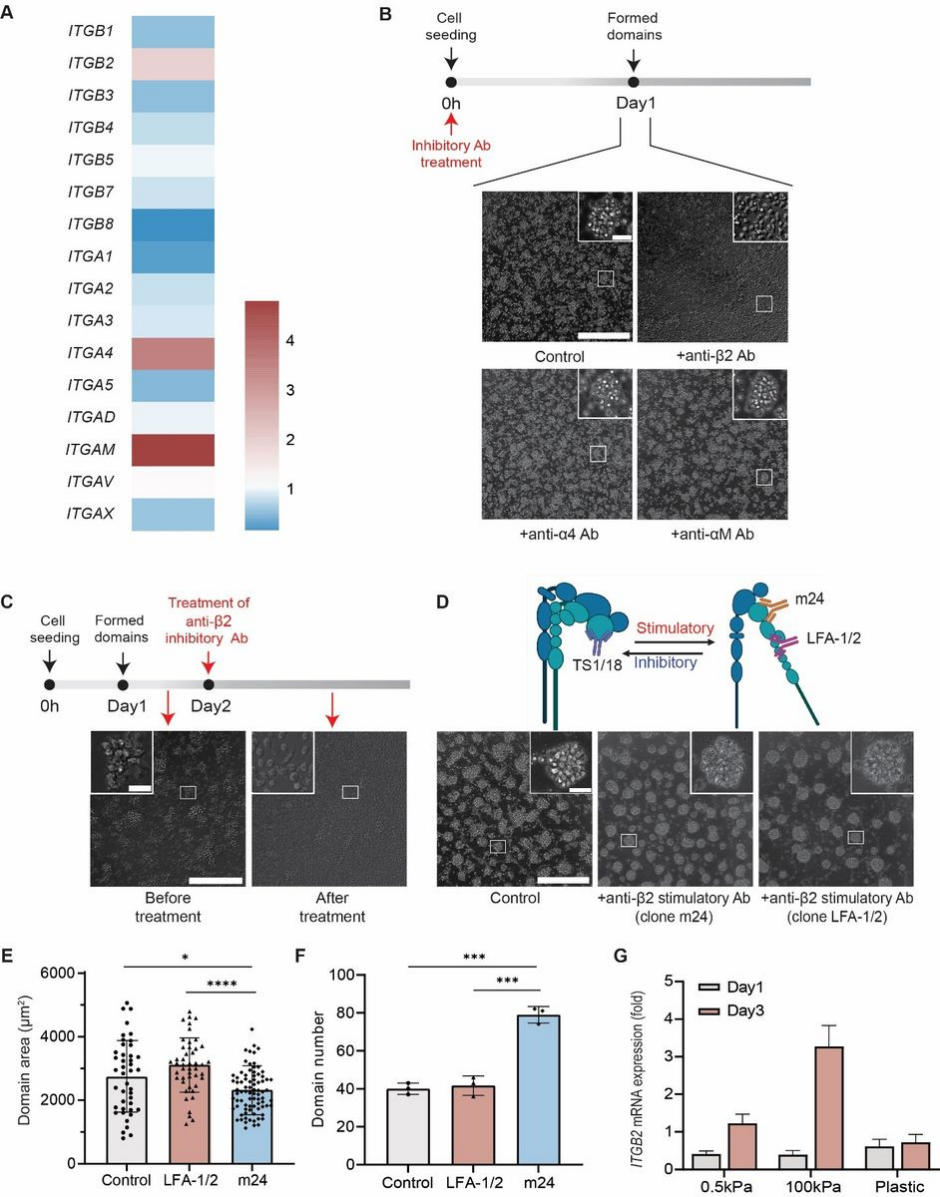
β2 integrin as a mediator of monocyte homotypic domain formation. (**A**) RT-qPCR analysis of cell surface integrins mRNA expression profile using cell samples collected on day1 and day 3 from collagen I-coated 0.5 kPa substrate. Relative fold changes of different integrins were measured relative to day 1, including upregulated *ITGB2* (integrin β2), *ITGA4* (integrin α4) and *ITGAM* (integrin αM). (N=3 donors and n=3 wells for a total of 9 experiments per condition). (**B**) Representative images of domain formation when monocytes were treated with inhibitory antibodies against integrins β2, α4 and αM during seeding. (**C**) Representative images of reversible domain collapsing on day 2 after formation when treated with inhibitory anti-β2 antibody. (**D**) Cartoon showing different binding subunits of inhibitory (clone TS1/18) and stimulatory (clone m24 and LFA-1/2) anti-β2 antibodies. Representative images of induced domain formation when monocytes were treated with stimulatory anti-β2 antibodies. (**E-F**) Characterizations of domain areas and numbers of monocytes when treated with stimulatory (clone m24 and LFA-1/2) anti- β2 antibodies. (**G**) RT-qPCR analysis of *ITGB2* expression trend on three substrates: collagen I-coated 0.5 kPa, 100 kPa, and plastic. The mRNA expression trend of *ITGB2*correlated well with the domain development over 3-day period on different substrates. Relative fold changes in expression of different integrins were measured relative to day 0. (N=3, n=3). Phase images were taken using a 20x objective. Scale bars, 300 μm (insets, scale bar, 30 μm). Unpaired student’s t-test was used for statistical analysis. Results are presented as mean ± standard deviation.

Because focal adhesions are integrin-containing structures involved in the crosstalk between matrix and intracellular actin networks and previous work on the monocytic cell line U937 reported the involvement of LFA-1/VLA-4 in their intercellular adhesion on tissue culture plastic^42,43^, we hypothesized that integrins mediated domain formation of monocytes. We compared the expression of cell surface integrins for monocytes collected from 0.5 kPa substrate at different time points. Among tested integrins, we found that integrin α4, αM, and β2 exhibited increased expression by day 3, for which we observed well-defined stable domain patterns, compared to day 1 (onset of domain formation), suggesting their potential roles in initiating and maintaining the formation of these domains (Fig. 3A). To validate these findings, we treated monocytes immediately after seeding with inhibitory antibodies targeting these three upregulated integrins. Only the anti-β2 integrin antibody completely abrogated domain formation (Fig. 3B). Finally, the application of inhibitory anti-β2 integrin antibody after domains had formed eliminated these domains (Fig. 3C, Supplementary Video 3), which suggests that β2 integrin is a main mediator of domain formation and also shows that domain formation is reversible.

To further establish cause and effect, we examined the effect of stimulatory antibodies specific to β2 integrin (clones m24 and LFA-1/2) and found that these activation antibodies significantly accelerated the initiation of monocytes domain formation (Supplementary Video 4), resulting in highly organized patterns after overnight culture (Fig. 3D). Interestingly, when a stimulatory β2 integrin antibody was added, domain area showed a slight decrease while the number of domains showed a slight increase (Fig. 3, E and F), thanks to a decreased number of non-aggregated (single) monocytes on the substrates. In sum, the use of both inhibitory and stimulatory anti-β2 antibodies demonstrated the direct involvement of β2 integrin in the domain formation of monocytes on soft matrix.

Since monocyte domain formation and progression differed for the tested substrate stiffnesses, we hypothesized that the level of expression of β2 integrin on monocytes placed on different substrates would vary accordingly. RT-qPCR analysis was performed on samples collected on days 1 and 3 (relative fold changes normalized to day 0), representing the initialization stage and stabilization/collapse stage of domains on 0.5 kPa, 100 kPa, and plastic collagen-coated substrates. Despite a slight downregulation of β2 integrin on day 1, elevated mRNA expression of β2 integrin on 0.5 and 100 kPa substrates but not on plastic substrate on day 3 correlated well with the mechano-induced domain pattern maintenance shown in Fig. 1C (Fig. 3G).

### Monocytes secrete global inhibitors

**Figure 4:**
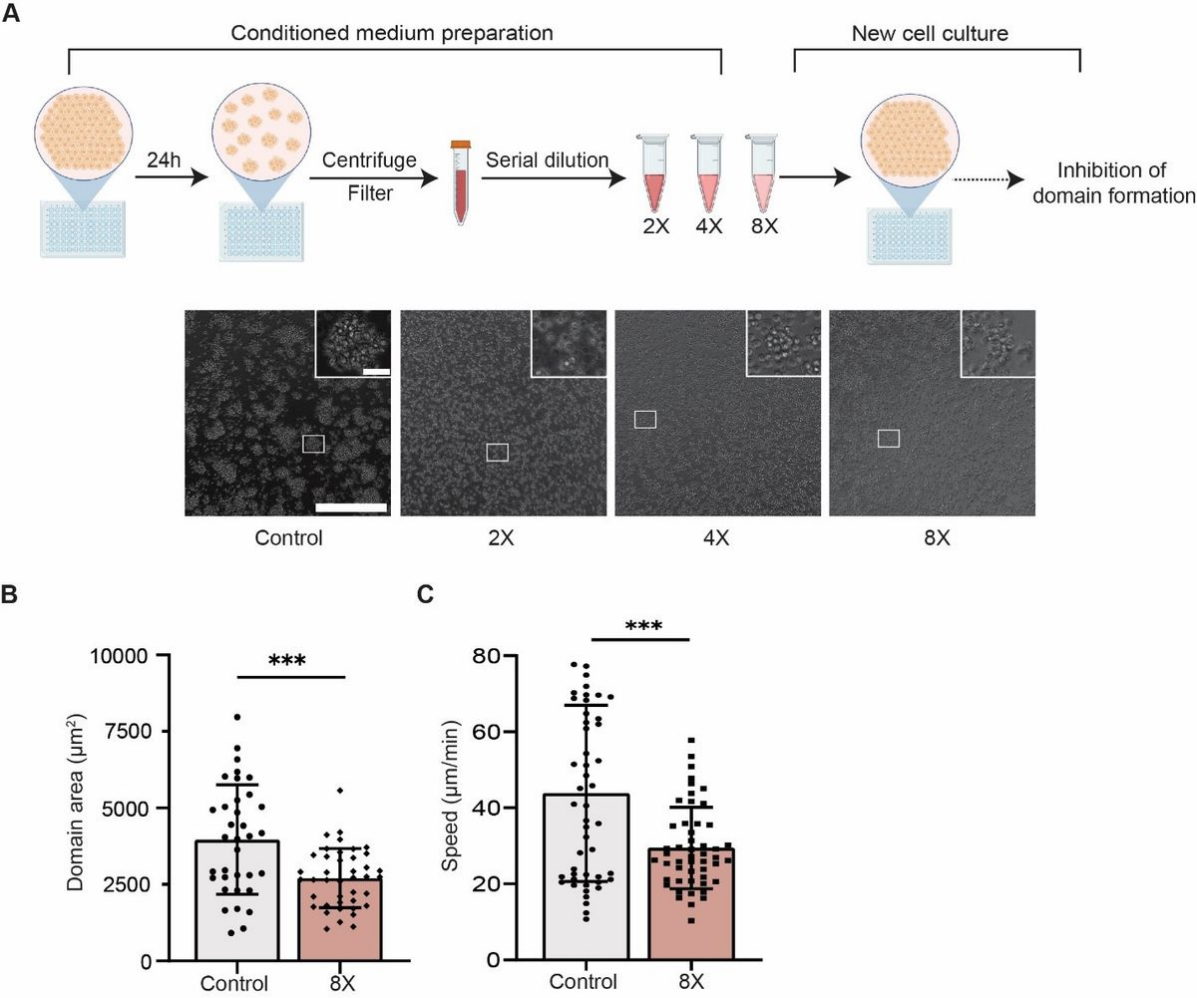
Conditioned media harvested from monocytic domains abrogated the onset of phase separation. (**A**) Conditioned medium harvested from steady state monocyte domains negatively influenced the domains formation initiation in a concentration-dependent manner. Only 8x diluted conditioned medium allowed for monocyte domains formation. (**B-C**) Decreased steady state domains area and cell speed decreased when monocytes were treated with 8x conditioned medium. Unpaired student’s t-test was used for statistical analysis. Results are presented as mean ± standard deviation. Scale bars, 300 μm (insets, scale bar, 30 μm).

Imortantly, global phase seperation of the monocyte/buffer system into a large monocyte-rich domain and a monocytes-poor domain did not occur, even for long obervation times (> 3 days). Since steady state domains patterns resulted from multiple domains that coalesce into a large one, we proposed the existence of monocyte-secreted inhibitory soluble factors that partially inhibited inter-cellular adhesion in the system, referred to “global inhibition”. To ascertain this hypothesis, conditioned medium from homotypically aggregated monocytes were harvested and applied on monocytes freshly seeded on a 0.5 kPa substrate. As depicted in Fig. 4A, monocytes treated with 2x or 4x diluted conditioned medium completely abolished domains formation, presumably due to the high concentration of inhibitory soluble factors secreted by monocytes during the initiation/incubation stage. The monocytes cultured in 8x diluted conditioned medium showed a delayed domains formation and featured significantly smaller domains size and decreased migration speed than control monocytes for overnight incubation (Fig. 4, B and C). As shown in Supplementary Fig. 3, secreted proteins from aggregated monocytes with a molecular weight around 110–130kDa might be the inhibitory soluble factors that drove the abrogation of domains formation in the presence of condition medium collected from aggregated cells. Identification of the specific inhibitory soluble molecules in the conditioned medium is beyond of the scope of this paper.

### A computational model describes domain formation and predicts key roles for cell migration and seeding density

This spontaneous, reversible, patterned homotypic aggregation of monocytes resembles the motility-induced phase separation (MIPS) of active matter^30,44,45^ and displays properties of pattern formation described by the Turing mechanism^46^. To further explore domain formation of monocytes, we developed a corresponding computational model based on the Cahn-Hilliard Eq. 3^6,37^. This model describes the process of phase separation by which the two components (monocytes + surrounding medium) of a binary fluid spontaneously separate and form domains that are pure in each component. Inhibitory factors were introduced to the system following the “global inhibition” theory that we propose and validated. Diffusion, cellular production and degradation of these soluble molecules were combined together to shape their distribution. Considering the relatively consistent initiation time of monocyte domain formation on 0.5 kPa substrate (Supplementary Fig. 1), we assumed an elevated affinity of cell adhesion molecules via mechano-sensing as the driving force of phase separation initialization, which we referred to as “local activation”.

The parameters and variables used for modeling the domain formation process are listed in Table 1. Due to the slow proliferation of human primary monocytes (Fig. 2B), cell growth was ignored when building up the model for simplification (characterization of domain area and number on day 3 of experiments were used to directly compare with steady state results of the simulation). The model consisted of individual cells in an environment that contains diffusible inhibitory molecules. We described the cells as adhesive spheres using an effective free energy function of the form $F=\int d\backslash varvecr\;\left[k_{4}\left(c_{1}\rho^{4}+c_{2}\rho^{3}+c_{3}\rho^{2}\right)+\frac{1}{2}k_{3}e^{-\alpha c}(\nabla \rho {)}^{2}\right]$. The first term of this equation is the prototypical bulk free energy of a hard-core particle with short range attraction, which roughly describes cells with mutual adhesion. The second term represents the interfacial energy between cells and the surrounding medium. We hypothesized that the interfacial energy was negatively influenced by inhibitory molecules, following a declining exponential function. Given the free energy function, the chemical potential driving motion is: $\mu =\frac{\delta F}{\delta \rho }$. Cells also migrate randomly, generating a diffusive behavior. The flux is then given by $j=-D_{1}\nabla \mu$ and the final equation for cell movement is described by: $\frac{\partial \rho }{\partial t}=-\nabla •j=D_{1}\nabla^{2}\mu$. The production and movement of inhibitory factors are described by a reaction-diffusion equation. The final governing equations for both cells and inhibitory factors are:

$$\frac{\partial \rho }{\partial t}=D_{1}\nabla^{2}\left[k_{4}\left(4c_{1}\rho^{3}+3c_{2}\rho^{2}+2c_{3}\rho \right)-k_{3}e^{-\alpha c}\left(-\alpha \nabla c\cdot \nabla \rho +\nabla^{2}\rho \right)\right]\;\;\frac{\partial c}{\partial t}=D_{2}\nabla^{2}c+k_{1}\rho^{2}-k_{2}c$$

Here,ρ and c are the cell density and concentration of inhibitory molecules, respectively. We solved the equations in a square region with side length l. In our model, we assumed no flux at the boundary for both cells (ρ) and inhibitory molecules (c). The initial cell density was set with a random small perturbation around a homogeneous state following a uniform distribution. The initial inhibitory molecule concentration was set as zero. To better match the domain area and number characterization results of simulations to experiments conditions, we further defined dimensionless parameters by setting characteristic length (L) and time (T) scales (Table 1). All the normalized variables and parameters in the equation are defined as: $\tilde{\rho }=\rho L^{2},\tilde{c}=cL^{2},\tilde{t}=\frac{t}{T},\backslash \tilde{varvec}x=\frac{\backslash varvecx}{L},\tilde{c_{1}}=\frac{c_{1}}{L^{4}},\tilde{c_{2}}=\frac{c_{2}}{L^{2}},\tilde{c_{3}}=c_{3},\tilde{D_{1}}=\frac{D_{1}T}{L^{2}},\tilde{\alpha }=\frac{\alpha }{L^{2}},\tilde{k_{4}}=k_{4},\tilde{k_{3}}=\frac{k_{3}}{L^{2}},\tilde{D_{2}}=\frac{D_{2}T}{L^{2}},\tilde{k_{1}}=\frac{k_{1}T}{L^{2}},\tilde{k_{2}}=k_{2}T$. The dimensionless parameters were fixed at base values (Table 1) in all simulations if not specified. The initial cell density was set as $\rho_{i}(\backslash \tilde{varvec}x)∼(0.7,0.8)$ if not specified. In all simulations, the length and time scales are set as: L=8μm,T=0.012h.

**Fig. 5:**
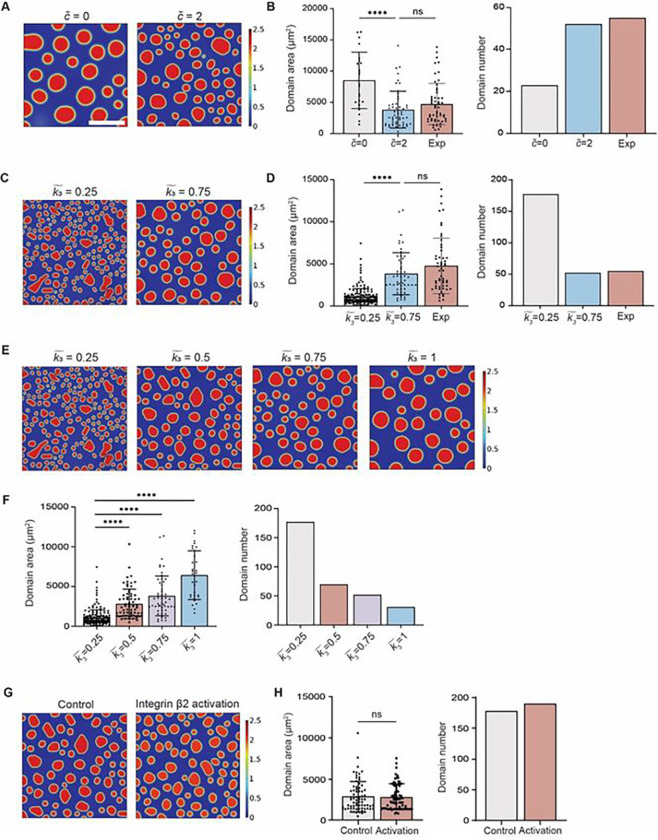
Validation of “global inhibition, local activation” in the simulation model via tuning of single parameters. (**A**) Effect of inhibitory factors on domain formation simulation at constant concentration of $\tilde{c}=0$ and $\tilde{c}=2$ (no production and degradation of inhibitory factors was considered). (**B**) Mean domain area decreased while domain number increased as the concentration of inhibitory factor increased, which matched the experimental characterization results. (**C**) Effect of interfacial energy coefficient on domain formation simulation at $\tilde{k_{3}}=0.25$ and $\tilde{k_{3}}=0.75$. (**D**) Increase in mean domain area and decrease in domain number to similar level of experimental characterization results was observed when higher interfacial energy coefficient was applied. (**E-F**) Increase of interfacial energy coefficient $\left(\tilde{k_{3}}\right)$ along with other key parameters fixed resulted in a monotonic increase in domain area and decrease in domain number. (**G-H**) By increasing interfacial energy coefficient $\left(\tilde{k_{3}}\right)$ and adjusting the bulk free energy coefficients $\left(\tilde{c_{1}},\tilde{c_{2}},\tilde{c_{3}}\right)$ to (0.1245, −0.5269, 0.5880), the mean domain area remained unchanged while domain number increases, which is consistent with experiment findings. In the simulation, all parameters except $\tilde{k_{3}},$ $\tilde{c_{1}},$ $\tilde{c_{2}},$ $\tilde{c_{3}}$ were fixed. Results were obtained at t=24h, representing the steady state. Unpaired student’s t-test was used for statistical analysis. Results are presented as mean ± standard deviation. Scale bar =300 μm.

To validate the “global inhibition” theory that we proposed, we first simplified the model by setting parameters $\tilde{k_{1}},\;\tilde{k_{2}}$ as 0 to ignore the production and degradation of the inhibitory diffusible factors. As shown in Fig. 5A–B, with all other parameters fixed, when no inhibitory factors existed in the system ($\tilde{c}=0$), scattered domains eventually merged into larger domains at steady state, which increased the mean domain area while decreased the domain number compared to scenario where inhibitory factors were set at a constant concentration $\left(\tilde{c}=2\right)$ across the whole simulation. Without inhibitory factors, domain area reached 8543 μm^2^ at steady state, which is significantly larger than the experimental domain area value at day 3 of 4771 μm^2^ (Fig. 1C). Similarly, when tuning up the coefficient of interfacial energy ${\tilde{k}}_{3}$ that reflects higher intercellular adhesion activity to simulate the “local activation” (production, degradation of inhibitory factors included), the mean domains area increased to 3809 μm^2^ and decreased the number of domains, which matched observed experimental values (Fig. 5, C and D).

After identifying β2 integrin as the “local activation” factor in domains formation, we were set to recapitulate the observed β2 integrin stimulation phenomenon (Fig. 3D–F) in our proposed computational model. We first adjusted the interfacial energy coefficient $\tilde{k_{3}}$ while fixing all other parameters to prove the independence of one key parameter’s influence on the simulation results. As the β2 integrin activity increased, there was a monotonic increase in the domain area and a decrease in the number of domains at steady state (Fig. 5, E and F). Then by increasing the interfacial energy coefficient $\tilde{k_{3}}$ and adjusting accordingly the bulk free energy coefficients $\tilde{c_{1}},\;\tilde{c_{2}},\;\tilde{c_{3}}$ to simulate the experimental setting, a similar trend of unchanged domain area and increased domain number was achieved at steady state (Fig. 5, G and H).

Taken together, we successfully built a Cahn-Hilliard equation-based computational model that closely matched the observed homotypic domain formation.

**Fig. 6:**
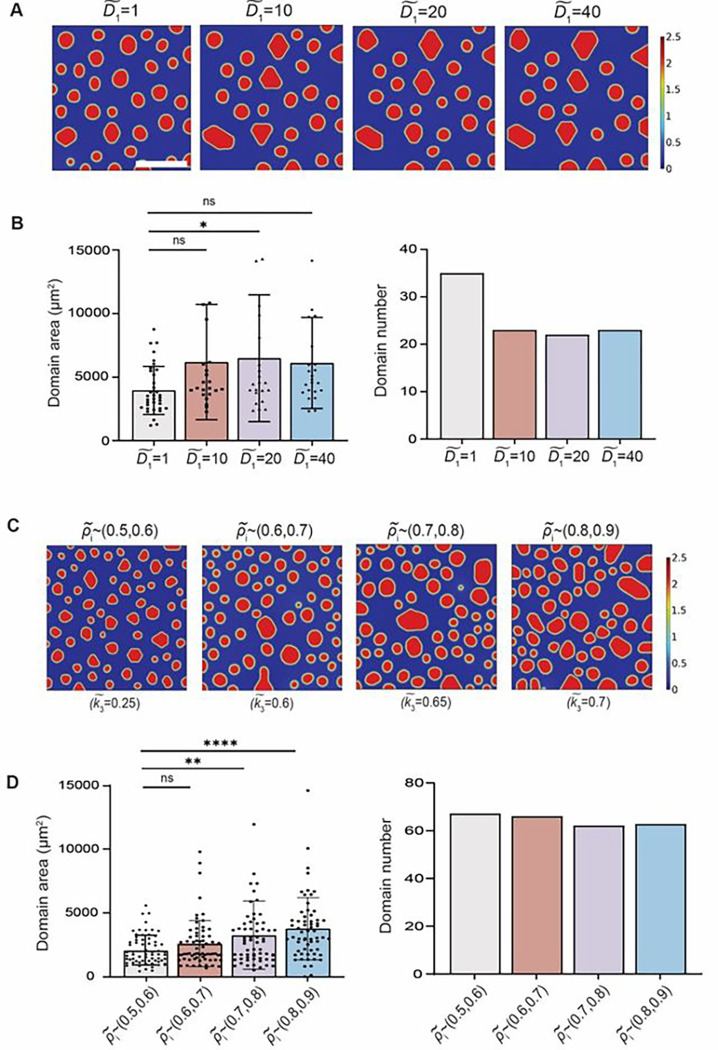
Model prediction on cell motility and seeding density’s effect on monocyte domain formation. (**A**) Simulation results illustrating steady state domain formation with monotonically increased diffusion coefficient $\tilde{D_{1}}$. All parameters except $\tilde{D_{1}}$ were fixed. (**B**) Characterization of domain area and domain number at different cell diffusion coefficient $\tilde{D_{1}}$. Domain area increased with decreasing domain number, which plateaued at $\tilde{D_{1}}=20$. (**C**) Simulation results illustrating steady state domain formation with monotonically increased initial cell density ${\tilde{\rho }}_{t}$. Coefficient of interfacial energy $\tilde{k_{3}}$ were changed correspondingly with different cell density ${\tilde{\rho }}_{4}$ to reflect the energy density as a function of cell density. (**D**) Characterization of domain area and domain number at different initial cell density $\tilde{\rho_{t}}$. All parameters except ${\tilde{\rho }}_{t}$ and coefficient of interfacial energy $\tilde{k_{3}}$ were fixed. Results were obtained at t=24h, representing the steady state. Unpaired student’s t-test was used for statistical analysis. Results are presented as mean ± standard deviation. Scale bar, 300 μm.

In addition to inhibitory soluble factor concentration $\tilde{c}$ and interfacial energy coefficient $\tilde{k_{3}}$, the cell diffusion coefficient ${\tilde{D}}_{1}$ (cell motility) and the initial cell density ${\tilde{\rho }}_{i}$ (seeding density) are two key parameters in the computational model that may affect the steady state simulation results. Here we provide predictions of how these two parameters may influence monocyte domain formation. When gradually tuning up the diffusivity coefficient of monocyte ${\tilde{D}}_{1}$, the model predicted that the steady state domain area increased while decreasing the number of domains (Fig. 6, A and B). Interestingly, a plateau was reached when ${\tilde{D}}_{1}$ became > 20, indicating that locally concentrated inhibitory molecules around formed domains were sufficient to block the merging of adjacent monocyte clusters, no matter how migratory the cells were. In terms of cell density, considering that the coefficients (e.g., interfacial energy coefficient ${\tilde{k}}_{3}$) in the free energy may be a function of cell seeding density^36^ and single-cell level integrin β2 expression was positively correlated with cell density (Supplementary Fig. 4), we increased the coefficient of interfacial energy $\tilde{k_{3}}$ correspondingly while increasing the initial cell density ${\tilde{\rho }}_{i}$. A monotonic increase of ${\tilde{\rho }}_{i}$ in the simulation resulted in increased domain area (Fig. 6C). Interestingly, when high cell density ${\tilde{\rho }}_{i}$ was applied, relatively consistent domain numbers were observed at steady state, which indicates that cell density in a single domain remained constant.

In the following sections, we carried out additional experiments to verify the accuracy of these model predictions.

### Influence of random and chemokine-directed cell motility on domain formation

Just as motility-induced phase separation of active matters^44,45^, the formation of domains observed in our study was driven by the active movement of monocytes on the substrates. Previous studies have categorized immune cell migration into two distinct modes: chemotaxis and random migration^47^. To understand whether the motility of monocytes could influence the domain formation process and whether the trend matched the model’s prediction, we treated monocytes with different inhibitors targeting either basal migration or chemotaxis pathways.

**Fig. 7:**
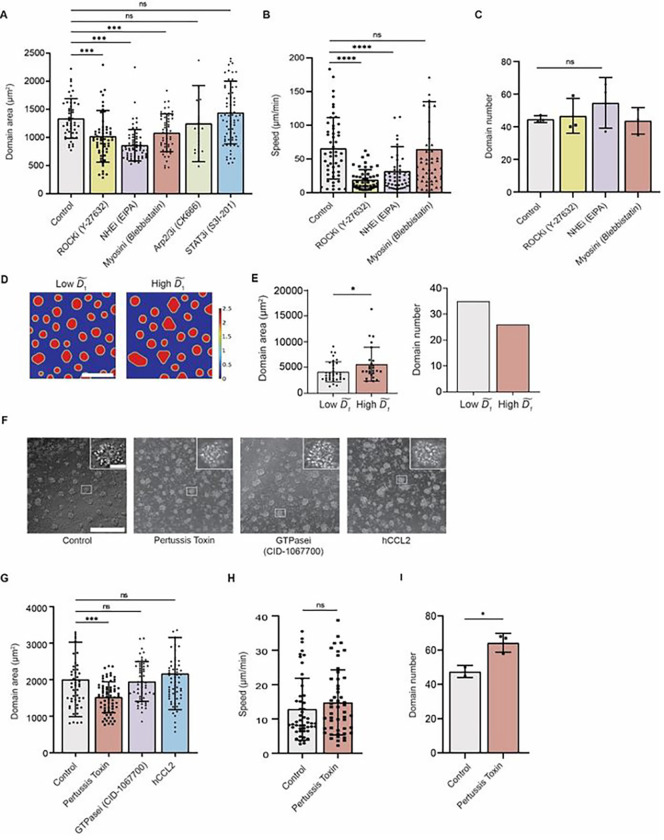
Modulation of monocyte homotypic domain formation by cell motility. (**A**) Monocytes seeded on 0.5 kPa substrate were treated with 20 μM ROCK inhibitor Y-27632, 100 μM Arp inhibitor CK666, 100 μM STAT3 inhibitor S3I-201, 10 μM Blebbistatin, and 10 μM EIPA. The average domain area significantly decreased following Y-27632, blebbistatin, and EIPA treatments. (**B**) Y27632 and EIPA treated monocytes showed significantly reduced cell motility. (**C**) No significant differences existed in domain numbers of Y27632, EIPA and Blebbistation treated conditions compared to control. Increased domain number when treated with EIPA was observed, though. (**D**) Representative simulation results comparing cells with low and high motility. In the simulation, all parameters were fixed except $\tilde{D_{1}}$. (**E**) Domain area decreased in the low cell motility group, while low cell motility led to an increase in domain number. (**F**) 20x representative images on monocyte domain formation when treated with complete medium, pertusis toxin, GTPasei and hCCL2, respectively. (**G**) Evaluation of domain area in the presence of three molecules targeting monocyte chemotaxis. Monocytes on 0.5 kPa substrate treated with pertussis toxin formed significantly smaller domains compared to the control group. (**H**) Cell motility did not significantly decrease upon pertussis toxin treatment. (**I**) Pertussis toxin treated monocytes showed increased domain number. Unpaired student’s t-test was used for statistical analysis. Results are presented as mean ± standard deviation. Scale bar = 300 μm for both experimental phase images and simulations (insets, scale bar, 30 μm).

Five inhibitors that targeted various cell migration pathways were selected to interfere with monocyte basal random migration. These include inhibitors for ROCK (Rho-associated protein kinase), Myosin, Arp2/3, STAT3, and NHE (Na+/H + ion exchanger)^48–50^.Inhibitions of ROCK, Myosin, and NHE all led to a significant decrease in the average domain area compared to the control group (Fig. 7A). Accordingly, cell motility was negatively influenced by ROCK and NHE inhibition (Fig. 7B). No significant difference was found in terms of domain number (Fig. 7C). Together, these results indicate that domain size is correlated with cell motility. Slower cell migration results in smaller domain size. Consistent with the random migration inhibitor experiments, decreased diffusion coefficient ${\tilde{D}}_{1}$ resulted in inadequate domain development in simulation with more domains with smaller area (Fig. 7, D and E).

To further validate the computational model in which diffusion of monocytes is considered as the sole driving force for domain formation instead of chemotaxis, we employed pertussis toxin (general chemotaxis inhibitor), CID-1067700 (a pan-GTPase inhibitor) and hCCL2 (block CCL2-CCR2 axis) to target monocyte chemotaxis. For these three treatments, monocytes were only found to aggregate into significantly smaller size (1522 ± 425 μm^2^) under the influence of pertussis toxin compared to the non-treated control group (2007 ± 1020μm^2^) (Fig. 7, F and G), which also resulted in higher domain number (Fig. 7I). However, pertussis toxin treatment did not significantly reduce cell movement compared to the control groups (Fig. 7H). These findings suggest that chemotaxis may not be the driving force behind monocyte aggregation in the domain formation process and diffusion along is sufficient to describe monocyte motion in the model.

### Onset of domain formation depends on cell seeding density

**Fig. 8:**
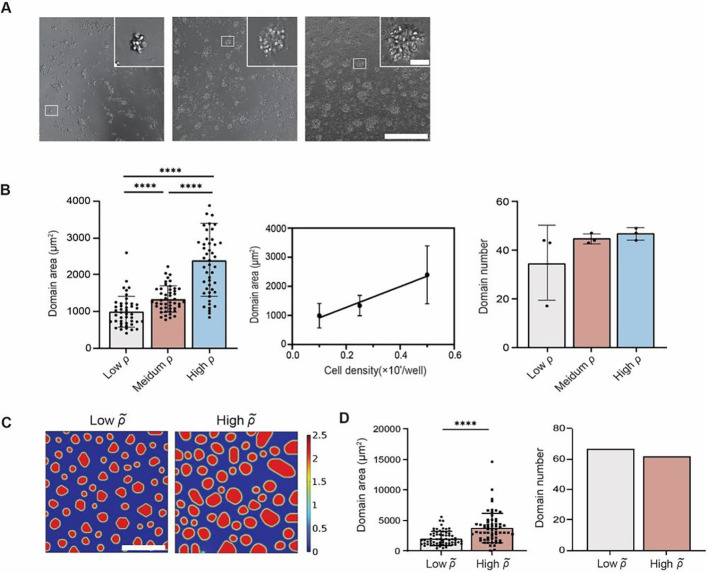
Monocyte homotypic domain formation was modulated by local seeding density. Three cell seeding densities (50,000, 25,000, 10,000 cells/well) representing high, medium, and low density were used. (**A**) Representative 20x images of three cell seeding density captured on day 1. (**B**) The averaged domain area on day 1 exhibited a linear correlation with cell seeding density. (**C**) Representative simulation results of low- and high-density groups $\left(\tilde{\rho_{l}}∼U(0.5,0.6)\right.$ and ${\tilde{\rho }}_{i}∼U(0.8,0.9)$). All parameters except seeding cell density $\tilde{\rho_{l}}$ and interfacial energy coefficient $\tilde{k_{3}}$ were fixed. (**D**) Significant increase of domain area was observed when comparing high seeding density group condition to low seeding density condition, while the high-density group exhibited a slight decrease in domain number. Results were obtained at t=24h, representing the steady state. Unpaired student’s t-test was used for statistical analysis. Results are presented as mean ± standard deviation. Scale bar = 300 μm for both experimental phase images and simulations (insets, scale bar = 30 μm).

To eliminate the possibility that monocyte domain formation was caused by excessive cells in each well, we adjusted the initial cell seeding density in each well. Three different seeding densities − 50,000 cells/well, 25,000 cells/well, and 10,000 cells/well - were used, which we refer to as high, medium, and low densities. On day 1, we observed that monocytes formed larger domains (2396 ± 996 μm^2^) at a high cell density compared with the low density (992 ± 423 μm^2^) (Fig. 8A). A linear correlation between the average monocyte domain area and the cell seeding density was observed (Fig. 8B). However, despite the variations in initial cell density, we consistently observed the initiation of domain formation across all three density groups (Supplementary Video 5). This suggests that the process of domain formation is independent of the initial monocyte seeding density, as evidenced by the formation of domains even in the low-density group where cells were initially spaced far apart.

Bumping up the seeding cell density from ${\tilde{\rho }}_{i}∼(0.5,0.6)$ to ${\tilde{\rho }}_{i}∼(0.8,0.9)$ in our computational model to mimic the low and high cell densities (25,000 *vs*. 50,000 cells/well) showed a significant increase in domain area (Fig. 8, C and D). Decreased domain number was observed, but the deviation wasn’t large. In general, the simulation results matched up well with the experimental outcomes with few concerns that remain to be solved. For example, although the initial diffusible factors concentration $\tilde{c}$ was set at 0, higher seeding cell density ${\tilde{\rho }}_{i}$ introduced considerable amount of inhibitory soluble factors that might interfere with the steady state simulation results. A time-delayed introduction of inhibitory soluble factors may offer a better solution for future optimization of the model.

## Discussion

To fight against pathogens upon maturation, monocytes originating from the bone marrow move into the bloodstream and are recruited to tissues, encountering different microenvironments of different mechanical stiffness. In solid tumors, monocytes can aggregate into dynamic complex structures (“hot spots” illustrated in Fig. 1A). Here, we demonstrate that primary human monocytes respond to differences in matrix stiffness in a unique way among major types of immune cells. On an ECM-coated matrix of low stiffness, the ensuing enhanced expression of β2 integrin on the monocyte surface, which initiates intercellular adhesion, and the secretion of global inhibitors together produce local cellular phase separation, resulting in reversible patterned domain formation and long-term maintenance.

Biological implications of this work remain to be tested *in vivo*, but the biophysical mechanism presented in this paper suggests that, for tumor onset and progression, hot spots formation can be initiated in inflamed soft tissues (stiffness, 1kPa) containing precursor lesions, not after cancer cells and cancer-associated fibroblasts deposit and crosslink collagen and other extracellular matrix molecules^51^, rendering the tumor matrix much stiffer (> 25 kPa)^38^.

In this work, we referred to the monocyte domain formation as “living-cell-system phase separation”, but it is worth pointing out that “microphase separation” has been used in the past to describe isoporous membrane formation driven by amphiphilic block copolymer self-assembly^52,53^, biomolecular condensates^54^ such as bacterial ribonucleoprotein bodies (BR-bodies), RNAP^55^, FtsZ^56,57^ and bacterial microdomains, which are crucial to biofilm formation^58,59^. Commonly observed bacterial aggregates from *E. coli*^60^ and *N. gonorrhoeae*^61,62^ are mainly driven by chemotactic interactions and are irreversible at steady state when proliferation takes over for further domain development. This fundamentally differs from the observed monocyte homotypic aggregation, which is reversible and where random migration is a main driving force, while proliferation shows negligible effect on domain pattern formation. Additionally, the finite size of bacterial domains is often summarized as a consequence of bacterial kinetic slowdown (motility loss) instead of global inhibition induced by secreted factors. More recent studies have focused on eukaryotic cells, like *D. discoideum*, modeling their non-Turing domain formation by incorporating oxygen availability as long-range repulsion^63^. Nevertheless, proper experimental and computational models that reflect cellular level soluble factor secretion and genetic modification to bridge active matter phase separation theory with living-cell systems are still lacking.

Our functional studies rationale for monocytes to aggregate on matrices of physiological stiffness: enhanced survival accompanied with decreased phagocytic capability. This optimization scheme is unique to living cells, and different from active matter considered thus far^44,45^. Moreover, self-propelled colloidal particles neither secrete inhibitory molecules, nor actively modulate adhesion molecules on their surface, which are two key ingredients leading to our observed patterned aggregates. Still, the resemblance between this spontaneous, reversible, patterned aggregation of monocytes and the motility-induced phase separation (MIPS) of active matters motivated us to build a corresponding computational model based on the Cahn-Hilliard equation combined with Turing model, which reflects the secretion of inhibitory soluble factors and changes in integrin gene expression to bridge active matter phase separation theory with living-cell systems.

Future iterations of the model are required to improve the accuracy of the simulations. For example, in contrast to our current simplification that cells produce inhibitory soluble factors at a constant rate from the beginning, time-delayed production of inhibitory factors based on the separation level (e.g., the characteristic distance between adjacent domains) can be applied so that high cell density does not introduce extremely high level of inhibitory soluble factors that might interfere with the normal initialization of monocyte domain formation. In addition, since the model is based on an active colloid-like system, the cellular complexity and heterogeneity was not fully taken into consideration. Our model does not take into account that single-cell level β2 integrin expression is positively correlated with cell seeding density (Supplementary Fig. 4). Moreover, aggregated monocytes may exhibit a different secretomic profile compared to actively migrating cells and potential effects of layer-stacking domain structure on characteristic domain areas are yet left unsolved in the current iteration of our model, which urges the incorporation of equations describing related cellular functions.

## Methods

### Isolation and culture of human primary monocytes / B cells / NK cells / T cells

PBMCs were isolated from donors’ whole blood by Ficoll-paque PLUS (cytiva) density gradient centrifugation. Human primary classical monocytes (CD14^+^CD16^−^) were further isolated using the Classical Monocyte Isolation Kit (Miltenyi Biotec) by magnetic-activated cell sorting (MACS). Human CD14^+^CD16^−^ monocytes were resuspended in DMEM (Corning) supplemented with 4.5g/L glucose, 10% v/v heat-inactivated fetal bovine serum (Corning), and 1% v/v penicillin-streptomycin (Sigma). Monocytes were cultured at a density of 0.5×10^6^ cells per well and incubated at 37°C. 96-well culture plates, either standard tissue culture plastic coated with collagen I or Softwell 96-well plates (Matrigen) containing collagen I coated polyacrylamide gel with stiffness values of 0.5 and 100 kPa were used. Similarly, human primary B cells / NK cells / T cells were isolated from donor PBMCs using corresponding isolation kits (B cell -- STEMCELL 17954; NK cell -- STEMCELL 17955; T cell -- STEMCELL 17911) per manufacturer protocol. B cells were cultured in ImmunoCult-XF B cell base medium (STEMCELL) with B cell expansion supplement (STEMCELL). NK cells were cultured in ImmunoCult NK cell base medium (STEMCELL) with NK cell expansion supplement (STEMCELL). T cells were cultured in X-VIVO 15 medium (Lonza) with 10% v/v FBS (Corning), IL-2 (R&D) and CD3/CD28 T cell activator (STEMCELL). Macrophages were differentiated from negatively isolated human primary monocytes in macrophage attachment medium -- DMEM (Corning) supplemented with 4.5g/L glucose, 10% v/v heat-inactivated fetal bovine serum (Corning), 1% v/v penicillin-streptomycin (Sigma) and 50 ng/mL human recombinant M-CSF (R&D). Monocytes were seeded at a density of 2.5×10^6^ in each well of a standard 6-well tissue-culture plate (5×10^6^ monocytes per well) in macrophage attachment medium the day they were isolated from donor PBMCs. After 3 days, cells remaining in suspension were gently aspirated away and fresh macrophage attachment medium were added. Macrophages were ready to be harvested and seeded upon 7 days of differentiation, when they were regarded as M0 naïve macrophages.

### Collagen I coating of tissue-culture plates

Based on the previously described method, low-concentration Collagen I (Corning 354249) was diluted with 0.1% acetic acid to a final concentration of 100 μg/mL. 96-well plastic cell culture plates were washed with PBS 3 times before adding 32 μL diluted collagen I per well. After 1 h of incubation, excess liquid in each well was aspirated out carefully followed by PBS washing. Complete DMEM medium was used to condition the plate for 1 h before use.

### Antibody inhibition of monocyte cell surface integrins

To explore the cell surface molecule engaged in the domain formation process, 6.25 μg/mL blocking antibodies against Integrin β2 (clone TS1/18, Biolegend) and 6.25 μg/mL activation antibodies against Integrin β2 (clone m24 and LFA1/2, Biolegend) were added in cell culture medium at the start of the culture.

### Chemical Inhibition of monocyte migration

Isolated human CD14^+^CD16^−^ monocytes were incubated with various inhibitors upon seeding. Several inhibitors were used to interfere with the random migration of monocytes: 20 μM RhoA Kinase ROCK inhibitor Y-27632 (Selleckchem), 100 μM Arp inhibitor CK666 (Selleckchem), 100 μM STAT3 inhibitor S3I-201 (Selleckchem), 10 μM Blebbistatin for non-muscle myosin II inhibition (Millipore Sigma), and 10 μM EIPA (Millipore Sigma) to block the sodium-hydrogen exchanger (NHE). Monocytes were incubated with 2μg/mL Pertussis toxin (EMD Millipore Crop), 20 μM CID-1067700 (MedChemExpress), and 2 μg/mL recombinant human CCL2 (Biolegend).

### PrestoBlue cell viability assay

2x working solution of PrestoBlue was prepared by mixing 10x stock solution to complete DMEM medium at a ratio of 1:5. 100μL of medium was gently aspirated from each testing well of a 96-well plate (200 μL in total) and 100 μL of 2x working solution was added slowly to avoid any agitation to the monocyte domains. After 3 h of incubation in a 37°C, 5% CO_2_ incubator, the plate was wrapped in aluminum foil and transferred to SpectraMax M3 plate reader (Molecular Devices). Fluorescence signals were read at an excitation of 560 nm and emission of 590 nm.

### Flow cytometry

Cell samples were washed 3 times in PBS and resuspended at a concentration of 1 million per mL. Cell suspensions were blocked with Human TruStain FcX (Biolegend) for 15 min under room temperature. The antibody staining solution was then added and incubated at 4°C for 30 min. Antibodies used for cell labeling were as follows: APC anti-human CD18 (clone LFA-1/2, Biolegend), and Propidium Iodide solution (Biolegend). Wash cells and then resuspend cells in 350 μL FAC wash buffer (1X DPBS containing 5% FBS, 1mM EDTA). Immunofluorescence-stained cells were analyzed on a FACS Canto. Analysis was performed with Flowjo Software version 10.4. The cell surface marker mean fluorescence intensity of each sample was corrected with the mean fluorescence intensity measured for corresponding isotype control.

### Gene expression analysis using RT-qPCR

Total RNA from monocytes subjected to different stiffness conditions and culture time was isolated using RNeasy Micro Kit (QIAGEN). cDNA synthesis was performed using iScript cDNA Synthesis Kit (Bio-Rad). Real-time PCR reactions were set up using iTaq Universal SYBR Green Supermix (Bio-Rad) and were executed in a thermal cycler (CFX384^™^ Real-Time System, Bio-Rad). The primers designed for specific gene amplification are listed in Table 2. Relative quantitation was performed using the △△Ct method in CFX Manager software.

### Western Blot

Whole-cell protein lysates were prepared in clear sample buffer (0.5M Tris pH 6.8, 20% SDS, 50% glycerol in water). Total protein concentration was evaluated using the Micro BCA^™^ Protein Assay Kit (Thermo Scientific). Based on the calculation, the same amount of protein was loaded on 4–15% SDS-PAGE gels (Bio-Rad) and ran at a voltage of 180V under room temperature for electrophoresis. Protein bands were transferred to the PVDF membrane using the Trans-Blot Turbo system (Bio-Rad). Membranes were blocked in 5% dry milk in TBST for 60 min at room temperature and incubated with diluted primary antibodies. Membranes were incubated with secondary antibodies for 60 min at room temperature. Then the membrane was analyzed under the ChemiDoc^™^ XRS + imaging system (Bio-Rad). Images were analyzed using Image Lab software. The intensity of the targeted protein band was normalized using housekeeping protein. Primary and secondary antibodies were used as follows: GAPDH (14C10) Rabbit mAb, Integrin β−2 (D4N5Z) Rabbit mAb, Anti-Rabbit IgG HRP-linked Antibody. All the antibodies were purchased from Cell Signaling Technology.

### Conditioned medium preparation and treatment

A total of 50,000 cells were seeded into each well of the 0.5kPa 96-well plate. Conditioned medium was harvested from each well after 24h or 72h incubation. Harvested supernatant was centrifuged at 1000 rpm for 5min and passed through a 0.22-μm filter to eliminate any possible cells in it. Aliquots of conditioned media were stored in the − 80°C freezer until use. Different dilutions of conditioned media were prepared by mixing the conditioned media with fresh DMEM in different volume ratios for further monocyte culture on 0.5 kPa substrate.

### Ultra centrifugation and silver staining of conditioned medium

Conditioned media were harvested from monocytes seeded on different substrates at different timepoints and filtered through 0.22-μm PES filters (Genesee Scientific). To normalize the loading amounts of total proteins, Western blot on Tryp-LE detached cell were carried out on house-keeping protein GAPDH (14C10) to determine the relative cell numbers for different conditions (cell numbers were assumed to be positively correlated with GAPDH band intensity). Conditioned media were concentrated at the ratio of 30x using Amicon Ultra centrifugal filter units with 10k MWCO (Millipore Sigma). Concentrated conditioned media normalized to cell numbers were loaded on 4–15% SDS-PAGE gels (Bio-Rad) and ran at a voltage of 180 V under room temperature for electrophoresis. After breaking SDS-PAGE gels out from the cassette, silver staining was then carried out per manufacturer’s protocol using Pierce Silver Stain kit (Thermo Scientific).

### Live-cell staining and phagocytosis assay

Monocytes were labeled using Cell Tracker Red (CMTPX, Invitrogen) immediately before seeding. After days of incubation, cells inside and outside the domains were separated by gentle pipetting. These two parts of cells were seeded to a new plastic plate at a density of 10,000 cells/well. Carboxylate-modified polystyrene fluorescent beads (Sigma), 1 μm in diameter, were added to the cells at the concentration of 5 beads/cell. Fluorescence images were taken every 30 min for 6 h. The merged images were obtained to count the total bead number per cell.

### Live-cell imaging and cell tracking

Human Classical monocytes (CD14^+^CD16^−^) were seeded at 50,000 cells/well on a 96-well plate. Images were taken every 1 min for 6 h using Nikon Eclipse Ti2 equipped with a stage top incubator. Cell movements were analyzed using MetaMorph and MATLAB. To ensure the accuracy of manual tracking, > 50 cells in each field of view were analyzed.

### Statistical analysis

Graphpad Prism 9 software was used for statistical analysis. An unpaired two-tailed student’s t-test was performed to evaluate the statistical significance between the two groups. For column analysis of multiple conditions, ordinary one-way ANOVA was performed. Significant values were given in grades P < 0.05(*), P < 0.01(**), P < 0.001(***), P < 0.0001(****).

## Figures and Tables

**Table 1:** Parameters and variables of the computational model

Parameter &variable	Dimensionless form	Description	Base value (dimensionless)
ρ	$\tilde{\rho }=\rho L^{2}$	Cell density	/
c	$\tilde{c}=cL^{2}$	The concentration of inhibitory factor	/
$D_{1}$	$\tilde{D_{1}}=\frac{D_{1}T}{L^{2}}$	Diffusion coefficient of cells	1
$D_{2}$	$\tilde{D_{2}}=\frac{D_{2}T}{L^{2}}$	Diffusion coefficient of inhibitory factor	20
$k_{1}$	$\tilde{k_{1}}=\frac{k_{1}T}{L^{2}}$	Production coefficient of the inhibitory factor	1
$k_{2}$	$\tilde{k_{2}}=k_{2}T$	Degradation coefficient of the inhibitory factor	0.5
$k_{3}$	$\tilde{k_{3}}=\frac{k_{3}}{L^{2}}$	Coefficient of interfacial energy	0.5
$k_{4}$	$\tilde{k_{4}}=k_{4}$	Coefficient of bulk free energy	2.5
$c_{1}$	$\tilde{c_{1}}=c_{1}/L^{4}$	Coefficient in bulk free energy	0.1024
$c_{2}$	$\tilde{c_{2}}=c_{2}/L^{2}$	Coefficient in bulk free energy	−0.4551
$c_{3}$	$\tilde{c}=c_{3}$	Coefficient in bulk free energy	0.5333
α	$\tilde{\alpha }=\alpha /L^{2}$	Coefficient of “global inhibition”	0.5
l	$\tilde{l}=l/L$	Side length of the square region	100

**Table 2.** Primer pairs targeting cell surface integrins

Gene	Primer	Sequences
*ITGA1*	Forward	5'-T CAATGACTTTCAGCGGCCC-3'
	Reverse	5'-TGGCCAACTAACGGAGAACCA-3'
*ITGA2*	Forward	5'-CAAACCCAGCGCAACTACGG-3'
	Reverse	5'-ACTGAGCGCTAACACCAGCA-3'
*ITGA3*	Forward	5'-TACACGATGCAGGTAGGCAG C-3'
	Reverse	5'-G CAAGAACACCGCGCCCATA-3'
*ITGA4*	Forward	5'-CTGTTCGGCTACTCGGTCGT-3'
	Reverse	5'-CTGGCCGGGATTCTTTCCGA-3'
*ITGA5*	Forward	5'-TGCACCACCAATCACCCCAT-3'
	Reverse	5'-TGAAACACTCAGCCTCCGGG-3'
*ITGA6*	Forward	5'-CAGCCCTTCCAACCCAAGGA-3'
	Reverse	5'-CAGCTTGCGAGCCATTCTGG-3'
*ITGA7*	Forward	5'-CGGCCAACTGTGTGGTGTTC-3'
	Reverse	5'-TGTTGGCCCGGACAATCACT-3'
*ITGA8*	Forward	5'-CACTGGCACCGAGACGTTTG-3'
	Reverse	5'-AGGCACTCCGATGGCAATGT-3'
*ITGA9*	Forward	5'-TGTCCGACAGCGTGGTTCTT-3'
	Reverse	5'-ATGTTGATGGAGCCCGGGAG-3'
*ITGA10*	Forward	5'-AGAGTGGAGTCAGGCCCCAT-3'
	Reverse	5'-GACCATCCACACTTCGGCCA-3'
*ITGA11*	Forward	5'-CCATCTTCCTGGCACCCCAT-3'
	Reverse	5'-CCTCTCATCCATGGTGGCGT-3'
*ITGAX*	Forward	5'-ATTAAATGACATTGCATCGAAGCCC-3'
	Reverse	5'-GAACGGGGCCATCAGGTGT-3'
*ITGAD*	Forward	5'-GGCACTGACACCCTGTTTGC-3'
	Reverse	5'-GGACGATGGGATCCACCAGG-3'
*ITGAL*	Forward	5'-CCCAGCCAAGTCAGCGGAT-3'
	Reverse	5'-TGCCAAGCCATCCCCTTCAA-3'
*ITGAM*	Forward	5'-GCTATGACTGGGCTGGTGGA-3'
	Reverse	5'-TGGCGGCAGCATAACCCAA-3'
*ITGAE*	Forward	5'-CAGATCCTGGATGAGCGGCA-3'
	Reverse	5'-CGTGTGTCGTAGAGCAACGC-3'
*ITGAV*	Forward	5'-TTTACTGGCGAGCAGATGGCT-3'
	Reverse	5'-GCCATCAGAGCCACGATCCA-3'
*ITGA2B*	Forward	5'-ATCACAAGCGGGATCGCAGA-3'
	Reverse	5'-CCGAGTCGCAGCTTACGAGA-3'
*ITGB1*	Forward	5'-GGGCCAAATTGTGGGTGGTG-3'
	Reverse	5'-TGTCATCTGGAGGGCAACCC-3'
*ITGB2*	Forward	5'-TTCGTGGACAAGACCGTGCT-3'
	Reverse	5'-TCCTTGTTGGGGCATGGGTT-3'
*ITGB3*	Forward	5'-CCTGCTCTCAGTGATGGGGG-3'
	Reverse	5'-TCTGGCGCGTTCTTCCTCAA-3'
*ITGB4*	Forward	5'-AGCACCCACATGGACCAACA-3'
	Reverse	5'-CTAGCCCCTGCTCTGTGCAT-3'
*ITGB5*	Forward	5'-GACCTATGTCTGCGGCCTGT-3'
	Reverse	5'-CACGCTCTGGTTCTCCCCAT-3'
*ITGB6*	Forward	5'-AGCTTCCTTCAGCGTGACTGT-3'
	Reverse	5'-CCCAGCCCCACAGGCTTTAT-3'
*ITGB7*	Forward	5'-GGACGCCAAGATCCCATCCA-3'
	Reverse	5'-TGGGGTGTGAGAGGATGCAC-3'
*ITGB8*	Forward	5'-TCGGATGGCGAAAAGAGGCT-3'
	Reverse	5'-GGCCTAGTGAGGGGTGTTCC-3'
*ICAM1*	Forward	5'-CCGTGAATGTGCTCTCCCCC-3'
	Reverse	5'-GAGGCGTGGCTTGTGTGTTC-3'
*ICAM2*	Forward	5'-TGATCTGCTGTCCAGGATCGGA-3'
	Reverse	5'-GCTGGTTACAGGTGGTGCTG-3'
*ICAM3*	Forward	5'-TGGACATTGAGGCTGGGAGC-3'
	Reverse	5'-TCGGCTGCATAGACGTGAGG-3'
*ICAM4*	Forward	5'-CCCATTACACTGATGCTCGCTTG-3'
	Reverse	5'-ATCCCCCTTTACGCCTGGGA-3'
*CD14*	Forward	5'-GGAAGACTTATCGACCATGGAGC-3'
	Reverse	5'-TCACAAGGTTCTGGCGTGGT-3'
*CD23*	Forward	5'-CGCAGAAATCCCAGTCCACG-3'
	Reverse	5'-TGCTTGAAGCCCGTTCAGGT-3'
